# Reliability and Utility of Standard Gene Sequence Barcodes for the Identification and Differentiation of Cyst Nematodes of the Genus *Heterodera*

**DOI:** 10.2478/jofnem-2022-0024

**Published:** 2022-07-29

**Authors:** Daniel C. Huston, Manda Khudhir, Mike Hodda

**Affiliations:** 1 Australian National Insect Collection, National Research Collections Australia, CSIRO, Canberra, ACT 2601, Australia

**Keywords:** detection, diagnosis, genetics, molecular biology, systematics, taxonomy

## Abstract

Difficulties inherent in the morphological identification of cyst nematodes of the genus *Heterodera* Schmidt, 1871, an important lineage of plant parasites, has led to broad adoption of molecular methods for diagnosing and differentiating species. The pool of publicly available sequence data has grown significantly over the past few decades, and over half of all known species of *Heterodera* have been characterized using one or more molecular markers commonly employed in DNA barcoding (18S, internal transcribed spacer [ITS], 28S, *coxI*). But how reliable are these data and how useful are these four markers for differentiating species? We downloaded all 18S, ITS, 28S, and *coxI* gene sequences available on the National Center for Biotechnology Information (NCBI) database, GenBank, for all species of *Heterodera* for which data were available. Using a combination of sequence comparison and tree-based phylogenetic methods, we evaluated this dataset for erroneous or otherwise problematic sequences and examined the utility of each molecular marker for the delineation of species. Although we find the rate of obviously erroneous sequences to be low, all four molecular markers failed to differentiate between at least one species pair. Our results suggest that while a combination of multiple markers is best for species identification, the *coxI* marker shows the most utility for species differentiation and should be favored over 18S, ITS, and 28S, where resources are limited. Presently, less than half the valid species of *Heterodera* have a sequence of *coxI* available, and only a third have more than one sequence of this marker.

Diagnoses of important plant pests are increasingly reliant on molecular gene sequence data. This is especially the case for plant-parasitic nematodes, a group for which traditional taxonomic expertise is in decline (Coomans, 2002; Eyualem-Abebe *et al*., 2006; Oliveira *et al*., 2011). Collectively, plant-parasitic nematodes are estimated to cause up to 15% loss of the total global crop production, valued at over 100 billion USD annually (Koenning *et al*., 1999; Abad *et al*., 2008; Nicol *et al*., 2011; Singh *et al*., 2013, 2015; Phani *et al*., 2021). The great diversity and species richness of plant-parasitic nematodes and their broad range of host associations and life cycle strategies (e.g., Procter, 1984; Boag and Yeates, 1998; Siddiqi, 2000; Oliveira *et al*., 2011; Palomares-Rius *et al*., 2014; Salas *et al*., 2022) mean that knowledge of species-level biology and life history is often needed for effective control strategies. Therefore, the ability to distinguish between closely related species is of critical importance.

The tylenchid family Heteroderidae Filipjev & Schuurmans Stekhoven, 1941, includes seven genera of “cyst nematodes,” a monophyletic lineage of sedentary plant parasites united primarily by the form taken by adult females at the end of their life cycle, a hardened sac containing embryonated eggs (Luc *et al*., 1986; Baldwin, 1992; Subbotin *et al*., 2001, 2010a, 2010b; Bert *et al*., 2008). The largest genus, *Heterodera* Schmidt, 1871, comprises about 85 species, many of which are devastating pests of important crops, including cereals, legumes, vegetables, and a wide variety of other crops (Nicol *et al*., 2007; Subbotin *et al*., 2010a, 2010b; Toumi *et al*., 2013; Smiley *et al*., 2017). The taxonomy of *Heterodera* is complex and morphological identification can be difficult as adults are sexually dimorphic; differences between many species are subtle and multiple life cycle stages are often required for accurate species identification and delineation (Subbotin *et al*., 2010b). Furthermore, a few species cannot be distinguished from one another morphologically at any life cycle stage (e.g., Subbotin *et al*., 2002). Thus, molecular sequence data have become an important part of diagnoses in this group.

Molecular identification of species of *Heterodera* initially relied on polymerase chain reaction (PCR) restriction fragment length polymorphism profiles (PCR-RFLP) (Waeyenberge *et al*., 2009). This methodology has largely been superseded by DNA sequencing, with species being characterized primarily with four markers commonly employed in DNA barcoding: the small subunit ribosomal RNA (18S rDNA), internal transcribed spacer (ITS; comprising ITS1-5.8S-ITS2), large subunit ribosomal RNA (28S rDNA), and the mitochondrial cytochrome oxidase subunit one (*coxI*) gene regions (e.g., Szalanski *et al*., 1997; Subbotin *et al*., 2000, 2010a, 2010b, 2018; Ferris *et al*., 2004; Mundo-Ocampo *et al*., 2008; Escobar-Avila *et al*., 2018; Powers *et al*., 2019). The number of sequences for species of *Heterodera* uploaded to the public database GenBank has accumulated rapidly since the late 1990s to early 2000s when these data first began to become available (e.g., Ferris *et al*., 1993; Szalanski *et al*., 1997; Subbotin *et al*., 2000, 2001). There have been some reports, however, of species pairs which cannot be differentiated using sequences of one or more of these genes (Subbotin *et al*., 2000, 2001, 2018; Waeyenberge *et al*., 2009; Vovlas *et al*., 2015; Sekimoto *et al*., 2017; Escobar-Avila *et al*., 2018). As we move into the genomic era, with all its implications for more rapid and better identification methodologies, it seems pertinent to assess the accuracy and utility of the pool of barcoding gene sequences that have accumulated over the last few decades as these data will undoubtedly be incorporated into the next generation of molecular diagnostic tools. Here, we evaluate these barcoding data with aims to identify the availability of sequences for species of the genus as a whole and across regions, the reliability of these data in terms of erroneous or otherwise unreliable sequences, and the utility of the 18S, ITS, 28S, and *coxI* markers for accurate species identification and delineation.

## Materials and Methods

Analyses here are based on sequence data obtained from the NCBI public database, GenBank, up to 10 August 2021. We downloaded all available partial or complete sequences of the 18S rDNA, ITS, 28S rDNA, and *coxI* gene regions for all named species of *Heterodera*. We note that ITS is situated between the 18S and 28S genes and comprises the ITS1, 5.8S, and ITS2 genes; we considered a sequence as “ITS” if it included a partial fragment or complete sequence of one of the latter three genes. We searched for all valid species individually and also searched using junior synonyms. We created a database of these sequences where each line of data records the GenBank accession number, species, gene region, geographic collection information, and sequence author(s) or publication reference. In those cases where some of this information was not included with the sequence record on GenBank, we sought it from the publication listed and/or through web searches of scholarly literature aiming to identify if the GenBank accession number in question had been published or referenced. Where collection locality could not be determined and where possible, collection locality was inferred based on the institutional addresses of the author(s) listed in the respective sequence record. Base statistics were calculated in Microsoft Excel, and a map depicting the geographic spread of available sequences was generated using ggplot2 (Wickham, 2009) in R (https://www.R-project.org) and edited in Adobe Illustrator CS6.

### Assessment of sequence reliability

We evaluated whether publicly available sequence data of the four markers selected were reliably assigned to the correct species of *Heterodera*, by determining the number of sequences labeled as a particular species of *Heterodera*, which were clearly not, or could not reliably be determined to be that species. To do this, we constructed individual sequence files for each species/gene marker combination represented in our database and added a sequence of *Globodera pallida* (Stone, 1973) Behrens, 1975 and *Globodera rostochiensis* (Wollenweber, 1923) Skarbilovich, 1959 to each file to serve as outgroup taxa. Each sequence file was aligned using MUSCLE (Edgar, 2004) as implemented in MEGA X (Kumar *et al*., 2018), except where there were less than five sequences of a particular marker for a species. Alignments were not trimmed or restricted to specific regions of the gene under analysis. Neighbor-joining trees based on each alignment file were constructed in MEGA X with the following parameters: 100 bootstrap replications, the number of differences model, inclusion of substitutions and transversions, uniform rates among sites, and pairwise deletion of gaps and missing data. Trees were examined by eye for clear outliers and potentially problematic sequences. Problematic sequences were defined as those that diverged significantly from putative congeners in the neighbor-joining trees generated. Such sequences were added to a new database and evaluated through reexamination of alignments and comparison against GenBank using BLAST (Altschul *et al*., 1990) to determine the source of observed divergence. Where there were less than five sequences of a particular marker for a species, sequences were compared directly against the GenBank database using BLAST. Divergence from other sequences attributed to the same, and other species was recorded.

### Assessment of sequence utility

The second aim of our study was to assess the power of each of the four molecular markers for accurate delineation of species. For this, we first generated four new sequence files, each including all sequences of *Heterodera* for each respective marker as above. Problematic sequences identified in our initial assessment of reliability were excluded, and sequences of *G. pallida* and *G. rostochiensis* were added as outgroup taxa as above. Alignments for each dataset were constructed using MAFFT via the online service (Katoh *et al*., 2019) and examined by eye for additional problematic sequences which would impede tree-based phylogenetic methods; such sequences were added to the database of problematic sequences and removed from their respective sequence files. Sequences were then re-aligned. Neighbor-joining trees were constructed for each dataset in MEGA X as above and examined by eye for clades which included sequences of two or more putatively different species that were poorly or not differentiated from one another. New data files were created for the sequences from each of these ambiguous species pairs or groups with sequences of *Globodera* spp. added as outgroup taxa. These ambiguous sequence datasets were aligned using MUSCLE and neighbor-joining trees as above and maximum likelihood trees using 100 bootstrap replications, and the general time reversible model with uniform rates among sites were also computed in MEGA X. Intraspecific and interspecific variation were further examined using pairwise comparison tables generated in MEGA X.

### Data availability

Our sequence databases and associated data, along with sequence FASTAs, alignments, trees, and other files are all made publicly available on the Commonwealth Scientific and Industrial Research Organisation (CSIRO) Data Access Portal.

## Results

### General results

Our sequence database includes 2,737 entries, comprising 77, 1,723, 345, and 592 sequences of the 18S, ITS, 28S, and *coxI* gene regions, respectively. Of the 2,737 sequences in our database, only 1,669 (61%) could be associated with a paper published in academic or other technical literature. Altogether, sequences were available for 66% of valid species of *Heterodera* (57 out of 87, based on our count at the time of writing; see Li *et al*., 2020; Hodda, 2022). Eight species were represented by just a single sequence, and for seven of these, this was of the ITS gene region. Only 13 species had at least one sequence of each marker, and only 11 species had more than a single sequence of each marker (Table 1; Supplementary Table 2).

**Table 1:** Numbers of species of Heterodera for which one or more gene sequences of one or more molecular markers (18S, ITS, 28S, and coxI) are available on GenBank.

	Marker				
Species of Heterodera	18S	ITS	28S	coxI	All
Species with at least one sequence	18	55	36	38	13
Species with more than one sequence	13	38	22	29	11

**Table 2:** Species pairs of Heterodera which are inadequately or poorly distinguished from one another using one or more of the standard molecular markers evaluated (18S, ITS, 28S, coxI), including minimum base pair differences observed between sequences of problematic species pairs.

Species group	Species pair	Gene region	Minimum base pair difference	Delineation power	Notes
Avenae	*H. avenae–H. filipjevi*	18S	1	Inadequate	
Avenae	*H. avenae–H. hordecalis*	18S	4	Weak	*H. hordecalis* not monophyletic
					in NJ/ML analyses; potentially
					distinguishable from closely
					related species in isolated
					analyses.
Avenae	*H. avenae–H. mani*	18S	1	Inadequate	
Avenae	*H. filipjevi–H. hordecalis*	18S	1	Inadequate	
Avenae	*H. filipjevi–H. mani*	18S	2	Inadequate	
Avenae	*H. hordecalis–H. mani*	18S	3	Weak	See above note.
Avenae	*H. arenaria–H. avenae*	ITS	0	Inadequate	
Avenae	*H. avenae–H. pratensis*	ITS	0	Inadequate	
Avenae	*H. avenae–H. australis*	ITS	2	Weak	Although only two bp different,
					sequences of *H. australis* form
					clade with some sequences
					of “*H. avenae*” which have
					been shown to be *H. australis*
					(see Subbotin *et al*., 2018).
Avenae	*H. avenae–H. mani*	ITS	2	Weak	Despite only two bp difference,
					sequences of *H. mani* form
					monophyletic clade to
					exclusion of other closely
					related sequences.
Avenae	*H. aucklandica–H*.	28S	1	Inadequate	Only one 28S sequence of
	*avenae*				*H. aucklandica* available for
					comparison.
Avenae	*H. aucklandica–H*.	28S	1	Inadequate	Two 28S sequences of
	*hordecalis*				*H. hordecalis* 9 bp different
Avenae	*H. aucklandica–H*.	28S	1	Inadequate	Only one 28S sequence of
	*pratensis*				*H. pratensis* available for
					comparison.
Avenae	*H. avenae–H. pratensis*	28S	0	inadequate	See above note.
Avenae	*H. avenae–H. hordecalis*	28S	0	Inadequate	Two 28S sequences of
					*H. hordecalis* 9 bp different
Avenae	*H. arenaria–H. avenae*	*cox*1	2	Weak	Despite only two bp
					difference, *H. arenaria* forms
					monophyletic clade within
					*H. avenae* group.
Cyperi	*H. elachista–H. oryzae*	ITS	1	Inadequate	
Cyperi	*H. elachista–H. oryzae*	28S	0	Inadequate	
Goettingiana	*H. carotae–H. cruciferae*	ITS	1	Inadequate	
Goettingiana	*H. goettingiana–H*.	28S	0	Inadequate	Two 28S sequences of
	*microulae*				*H. goettingiana* are 19 bp
					different from one another; one
					potentially misidentified
Goettingiana	*H. carotae–H. urticae*	28S	2	Weak	Only one 28S sequence
					of *H. urticae* available for
					comparison.
Goettingiana	*H. carotae–H. cruciferae*	28S	2	Weak	Only one 28S sequence of
					*H. cruciferae* available for
					comparison (two on GenBank
					but one appears to actually be
					*H. schachtii*).
Goettingiana	*H. carotae–H. cruciferae*	*cox*1	1	Inadequate	Three *cox*1 sequences
					available for *H. cruciferae*, one
					15 bp different from others.
Schachtii	*H. betae–H. glycines*	18S	2	Weak	*H. glycines* forms
					monophyletic clade to
					exclusion of *H. betae*,
					*H. schachtii* & *H. trifolii* in ML,
					but not NJ, analyses.
Schachtii	*H. betae–H. schachtii*	18S	0	Inadequate	
Schachtii	*H. betae–H. trifolii*	18S	0	Inadequate	
Schachtii	*H. schachtii–H. glycines*	18S	2	Weak	See above note.
Schachtii	*H. schachtii–H. trifolii*	18S	0	Inadequate	
Schachtii	*H. trifolii–H. glycines*	18S	2	Weak	See above note.
Schachtii	*H. betae–H. daverti*	ITS	0	Inadequate	
Schachtii	*H. betae–H. schachtii*	ITS	0	Inadequate	
Schachtii	*H. betae–H. trifolii*	ITS	0	Inadequate	
Schachtii	*H. ciceri–H. schachtii*	ITS	2	Inadequate	
Schachtii	*H. ciceri–H. trifolii*	ITS	1	Inadequate	
Schachtii	*H. daverti–H. schacthii*	ITS	0	Inadequate	
Schachtii	*H. daverti–H. trifolii*	ITS	0	Inadequate	
Schachtii	*H. glycines–H*.	ITS	1	Inadequate	
	*medicaginis*				
Schachtii	*H. schachtii–H. trifolii*	ITS	0	Inadequate	
Schachtii	*H. betae–H. daverti*	28S	1	Inadequate	
Schachtii	*H. betae–H. schachtii*	28S	2	Inadequate	
Schachtii	*H. betae–H. trifolii*	28S	1	Inadequate	
Schachtii	*H. daverti–H. schachtii*	28S	3	Weak	
Schachtii	*H. daverti–H. trifolii*	28S	1	Inadequate	
Schachtii	*H. schachtii–H. trifolii*	28S	1	Inadequate	

**Figure 1:**
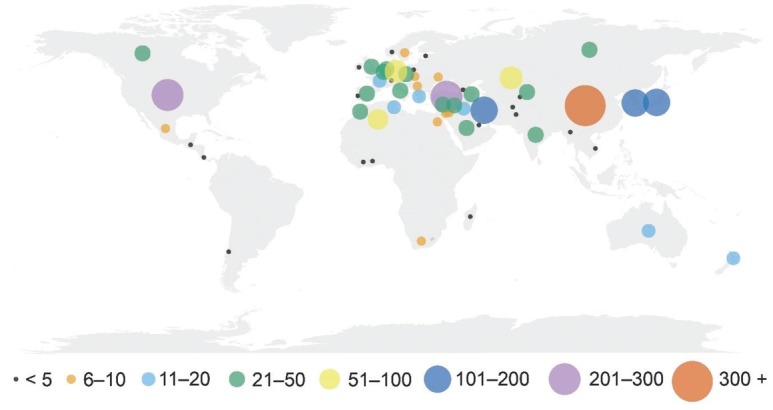
Geographic origin of 18S, ITS, 28S, and *coxI* gene sequences analyzed in the present study. The size of the circle reflects the total number of sequences of all markers centered on the country of origin.

Sequence data were attributed to specimens collected from 59 countries, from all continents except Antarctica (Fig. 1). More than half came from three countries: China (937), Turkey (272), and the USA (254), while 19 countries had less than five sequences attributed to them, including Afghanistan, Chile, Côte d’Ivoire (Ivory Coast), and Qatar, each with just one record (Supplementary Table 1). Notably, very few sequences originated from sub-Saharan Africa and only a single sequence originated from South America. Iran had sequences attributed to the most species (19), followed by the USA (18), China (16), and Germany (15) (Supplementary Table 2).

### Assessment of reliability

**Figure 2:**
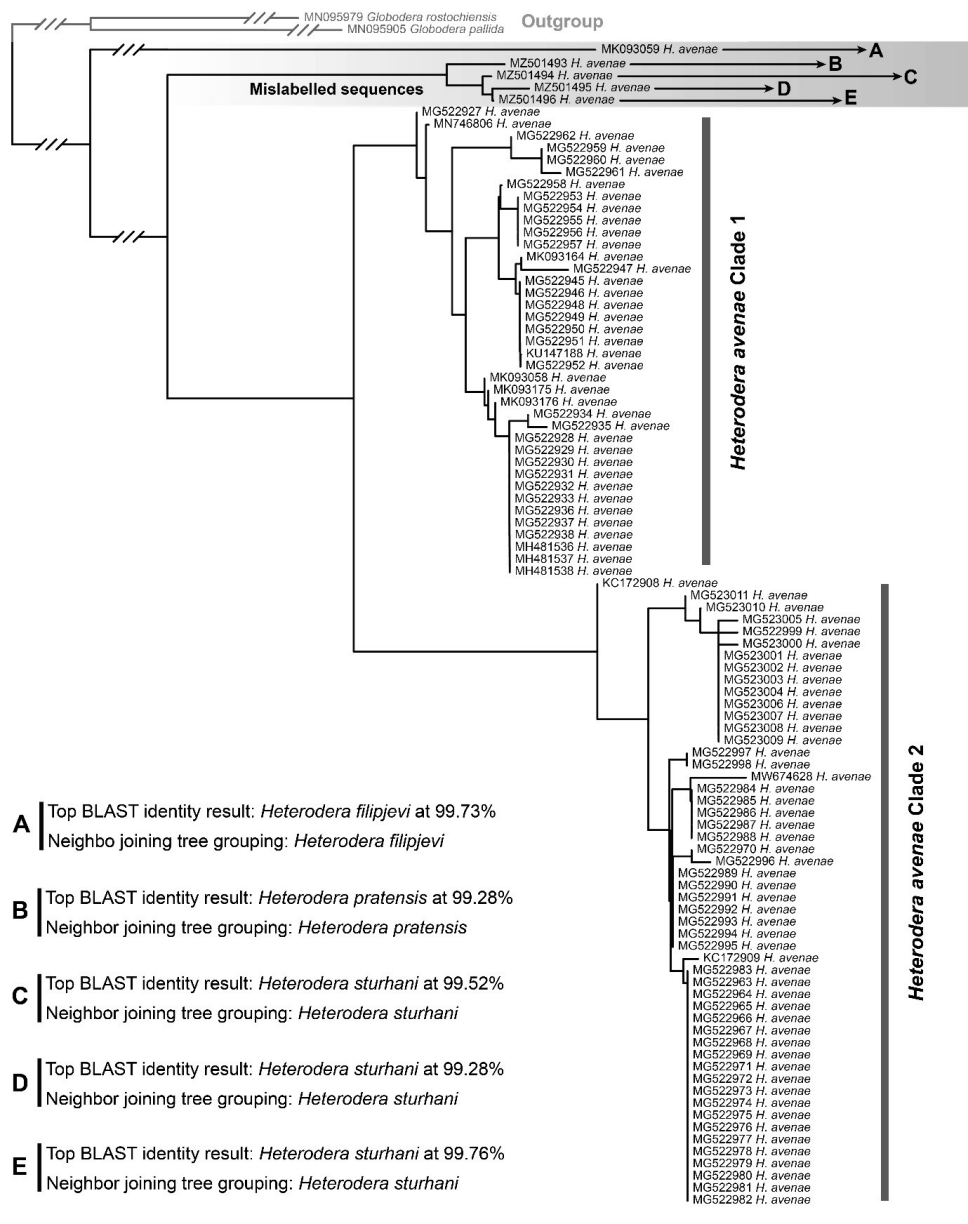
Neighbor-joining tree based on *coxI* sequences of *Heterodera* avenae from the NCBI database GenBank showing a clade of mislabeled sequences.

The overall rate of erroneous sequences was low, with only 55 of the total 2,737 sequences (2%) being detected as potentially problematic. Of these 55 sequences, 25 were accurate but had been uploaded running in the negative strand (3¢ to 5¢) and required reverse complementing before they could be aligned with congeners. The remaining 30 sequences were considered truly erroneous or otherwise unreliable. Issues appeared to include poor-quality sequencing results and/or sequence editing errors, sequences of *Heterodera* uploaded under the wrong species name (e.g., Fig. 2), misidentification of related nematodes as *Heterodera*, and clear contamination (including a sequence of a fungus and cucumber).

### Assessment of sequence utility

**Figure 3:**
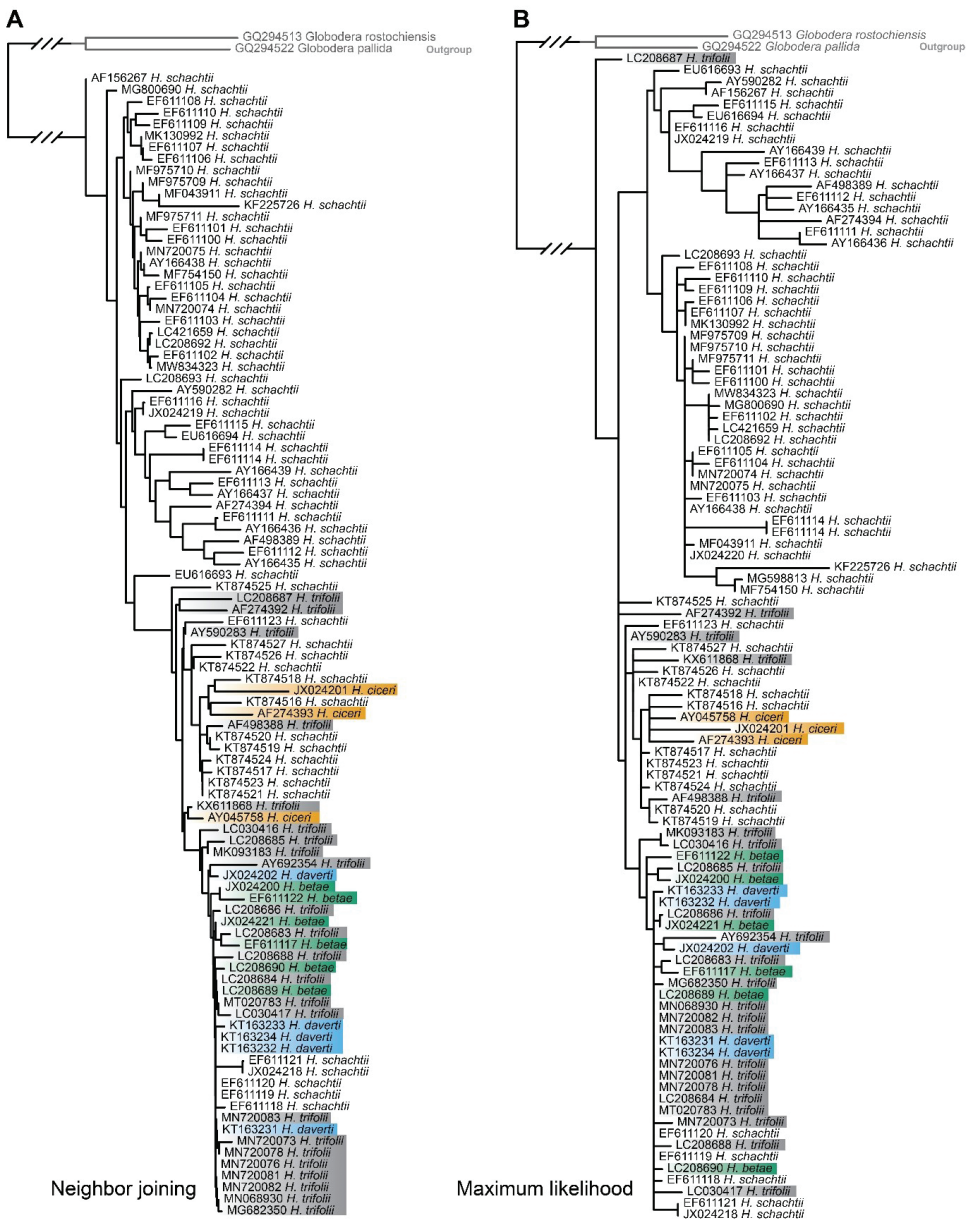
Neighbor joining (A) and maximum likelihood (B) trees derived from analyses of ITS gene sequences for the Schachtii species group showing multiple instances where species cannot be differentiated.

Each of the four molecular markers evaluated could not differentiate at least one species pair (Table 2). In many cases, putatively distinct species shared identical sequences in one or more of the markers evaluated (Table 2; Fig. 3). Many other comparisons included differences of only one or two base positions (bp), and these slight differences were generally inadequate for species delineation in tree-based methods and BLAST. Intraspecific genetic variation observed for some species of *Heterodera* (Table 3) spans the interspecific variation present within some species groups (e.g., Table 2). There were, however, some slight differences in sequences that were consistent enough to delineate species reliably. For example, despite having ITS sequences differing by only two bp from those of *H. avenae* Wollenweber, 1924, *H. mani* Mathews, 1971, consistently formed a monophyletic clade to the exclusion of other related species in tree-based methods.

**Table 3:** Intraspecific differences observed in the 18S, ITS, 28S, and *coxl* gene regions for those species of *Heterodera* for which more than one sequence of one or more of the respective gene regions are available, excluding problematic sequences.

	18S		ITS		28S		cox1	
	
Species	*n*	bp (%)	*n*	bp (%)	*n*	bp (%)	*n*	bp (%)
*Heterodera arenaria*			4	0–1; 0.50 (0–0.29; 0.15)			6	0–2; 2 (0–0.48; 0.48)
*Heterodera aucklandica*			3	0(0)			5	0–1; 0 (0–0.24; 0)
*Heterodera australis*			18	0–9; 3 (0–0.93; 0.31)			5	0(0)
*Heterodera avenae*	17	0–11; 1 (0–1.09; 0.06)	542	0–80; 8 (0–11.89; 0.89)	76	0–5; 0 (0–0.67; 0)	91	0–34; 9 (0–8.17; 2.40)
*Heterodera betae*	2	1 (0.06)	7	0–12; 3 (0–1.29; 0.37)	2	0(0)	2	0(0)
*Heterodera bifenestra*			3	2–10; 8 (0.20–1.02; 0.81)				
*Heterodera cajani*			12	0–12; 4 (0–1.18; 0.39)	13	0–11; 2 (0–1.46; 0.26)		
*Heterodera carotae*			13	0–13; 6 (0–1.35; 0.62)	8	0–6; 3 (0–0.83; 0.41)	17	0–20; 3 (0–4.77; 0.82)
*Heterodera ciceri*			3	7–14; 11 (0.74–1.49; 1.18)				
*Heterodera cruciferae*			11	0–10; 3 (0–1.21; 0.36)	2	52 (9.49)	3	1–16; 16 (0.24–4.01; 3.77)
*Heterodera cyperi*			2	0(0)				
*Heterodera daverti*			5	0–2; 1 (0–0.24; 0.11)			3	0–2; 2 (0–0.51; 0.51)
*Heterodera dunensis*	3	0(0)	3	0(0)	3	0(0)	8	0–1; 0 (0–0.28; 0)
*Heterodera elachista*	3	3–4; 3 (0.25–0.36; 0.36)	43	0–48; 11 (0–4.52; 1.05)	18	0–4; 1 (0–0.54; 0.15)	2	0(0)
*Heterodera fici*	2	2 (0.34)	8	0–3; 1 (0–0.31; 0.10)			9	0–2; 1 (0–0.47; 0.24)
*Heterodera filipjevi*	3	1–2; 1 (0.12–0.17; 0.17)	256	0–43; 2 (0–4.46; 0.21)	10	0–2; 1 (0–0.30; 0.13)	75	0–44; 10 (0–10.58; 2.42)
*Heterodera glycines*	6	0–1; 0 (0–0.16; 0)	319	0–112; 3 (0–11.89; 0.32)	56	0–43; 2 (0–5.79; 0.26)	81	0–9; 0 (0–1.04; 0)
*Heterodera goettingiana*	4	0–10; 5 (0–1.21; 0.59)	15	0–81; 37 (0–11.34; 5.08)	2	19 (2.92)	3	0–17; 17 (0–4.84; 4.84)
*Heterodera goldeni*			5	0–6; 2 (0–0.59; 0.23)				
*Heterodera guangdongensis*			3	0–3; 3 (0–0.26; 0.26)	7	1–6; 4 (0.15–0.91; 0.58)		
*Heterodera hordecalis*	2	5 (0.31)	37	0–68; 7 (0–8.89; 0.84)	2	9 (1.33)	10	0–47; 14 (0–11.4; 3.37)
*Heterodera humuli*			13	0–4; 1 (0–0.57; 0.17)			19	0–9; 2 (0–5.92; 0.59)
*Heterodera koreana*	3	1–22; 2 (0.17–1.27; 0.34)	29	0–28; 7 (0–3.08; 0.73)	48	0–21; 2 (0–2.88; 0.27)	44	0–18; 5 (0–4.57; 1.27)
*Heterodera latipons*			154	0–122; 13 (0–13.32; 1.46)	*2*	1 (0.15)	16	0–50; 27 (0–12.05; 6.52)
*Heterodera litoralis*							4	0(0)
*Heterodera mani*			6	1–9; 3 (0.10–0.93; 0.31)			6	0–1; 1 (0–0.27; 0.24)
*Heterodera medicaginis*			14	0–9; 3 (0–0.95; 0.42)			14	0–2; 0 (0–0.49; 0)
*Heterodera mediterranea*	*2*	1 (0.17)	6	1–9; 7 (0.11–0.95; 0.74)	*2*	3 (0.38)		
*Heterodera mothi*			2	3 (0.30)			2	2 (0.44)
*Heterodera orientalis*			5	6–18; 12 (0.66–1.94; 1.28)				
*Heterodera pratensis*			13	0–10; 6 (0–1.08; 0.62)			20	0–8; 2 (0–1.93; 0.53)
*Heterodera ripae*			5	0–4; 2 (0–0.46; 0.22)			19	0–11; 1 (0–2.59; 0.24)
*Heterodera sacchari*			3	0–1; 1 (0–0.09; 0.09)				
*Heterodera salixophila*			5	0–20; 15 (0–2.10; 1.58)	*2*	1 (0.15)	16	0–18; 2 (0–4.57; 0.51)
*Heterodera schachtii*	9	1–9; 4 (0.06–0.99; 0.31)	64	0–31; 12 (0–3.04; 1.29)	20	0–4; 1 (0–0.58; 0.13)	38	0–7; 1 (0–1.88; 0.12)
*Heterodera sojae*			5	0–12; 4.5 (0–1.36; 1.03)	10	0–8; 4 (0–1.06; 0.53)	12	0–6; 1 (0–1.59; 0.24)
*Heterodera sturhani*							7	0–3; 0 (0–0.72; 0)
*Heterodera trifolii*	5	0–8; 3 (0–0.47; 0.29)	23	0–14; 3 (0–1.48; 0.43)	19	0–1; 0 (0–0.15; 0)	26	0–5; 0 (0–1.24; 0)
*Heterodera ustinovi*			5	1–8; 3.5 (0.10–0.88; 0.36)			5	0–1; 1 (0–0.24; 0.24)
*Heterodera zeae*			16	0–39; 14 (0–5.65; 2.19)	4	0–2; 1 (0–0.31; 0.15)		

Intraspecific differences are presented in the format “base pair difference range; median of base pair difference range (percent difference range; median of percent difference range).” Medians indicated only in cases of three or more comparisons; *n =* the number of sequences compared.

Fifteen species pairs could not be reliably delineated using both the ITS and 28S gene regions, followed by 12 species pair issues in the 18S gene region and just two in the *coxI* region (Table 2). Notably, several species pairs could not be distinguished across multiple markers. For example, *H. avenae* shares some identical ITS and 28S gene sequences with *H. pratensis* Gäbler, Sturhan, Subbotin & Rumpenhorst, 2000. *Heterodera schachtii* A. Schmidt, 1871 shares some identical sequences with *H. betae* Wouts, Rumpenhorst & Sturhan, 2001, and *H. trifolii* Goffart, 1932 in the 18S and ITS gene regions and differs from these species by only one and two bp in the 28S gene region, respectively. Sequences of *coxI* had the most utility for distinguishing between species, with just one case of a weak, and one case of an inadequate species pair delineation; these notable cases are discussed further below.

## Discussion

Our analyses indicate that several of the most commonly employed molecular markers for the characterization of species of *Heterodera* lack the necessary resolution for distinguishing between some species, including a number of plant pests of significant global biosecurity concern. Among those for which molecular identification issues were detected, 13 species (*H. avenae*, *H. carotae* Jones, 1950a, *H. ciceri* Vovlas, Greco & Di Vito, 1985, *H. cruciferae* Franklin, 1945, *H. daverti* Wouts & Sturhan, 1978, *H. elachista* Ohshima, 1974, *H. filipjevi* (Madzhidov, 1981) Stelter, 1984, *H. glycines* Ichinohe, 1952, *H. goettingiana* Liebscher, 1892, *H. hordecalis* Andersson, 1975, *H. oryzae* Luc & Brizuela, 1961, *H. schachtii* and *H. trifolii*) are listed as regulated pests in at least one country (Singh *et al*., 2013). This has numerous implications for routine molecular diagnoses of species of *Heterodera*, such as the potential for confusing a major pest with a minor or non-pest species. For example, *H. avenae* is a major pest of cereals in temperate regions and causes significant annual yield losses throughout its range (e.g., Meagher, 1982; Smiley *et al*., 2005; Nicol and Rivoal, 2008), but our analyses showed that it could not be reliably distinguished from *H. arenaria* Cooper, 1955 or *H. pratensis* using the most commonly employed molecular marker, ITS. Both latter species parasitize non-crop grasses (Subbotin *et al*., 2018); thus, there is potential for confusing *H. avenae* with one of these non-pest species. Such cases of mistaken identity could result in misdiagnosed infestations and novel incursions. In addition, mixed infestations of some closely related species which parasitize similar crops, such as members of the Schachtii group, may go undetected.

Although a combination of morphological data and sequences from multiple gene regions is best for identification of species of *Heterodera*, our results suggest that where resources or expertise are limited, the *coxI* region should be favored over 18S, ITS, and 28S for basic diagnostic purposes. It is significant that a number of economically important species of *Heterodera* cannot be delineated using ITS; as to date this marker has been used more extensively than any other for both identification and phylogenetic Purposes (e.g., Subbotin *et al*., 2001, 2017; Tanha Maafi *et al*., 2003). Furthermore, more species of *Heterodera* have been characterized with ITS than any other marker, and for many species, this is the only gene region for which sequences are available. Thus, when developing molecular diagnostic tests for plant-parasitic nematodes like *Heterodera* spp., the trade-off between delineation power and species coverage needs careful consideration. There is currently a great amount of interest in employing high-throughput sequencing methods, such as metabarcoding and eDNA, for detection and monitoring of pest species (e.g., Abdelfattah *et al*., 2018; Valentin *et al*., 2018; Ruppert *et al*., 2019; Hardulak *et al*., 2020; Young *et al*., 2021). Many metabarcoding studies have utilized short fragments of nuclear genes such as 18S for species detection and identifications (e.g., Macheriotou *et al*., 2019; Ruppert *et al*., 2019; Giebner *et al*., 2020), but for *Heterodera*, fragments of the nuclear genes tested here lack the power to distinguish between all species. Those using such tools will need to be aware of the characteristics and shortcomings of the marker(s) employed and may need to follow-up species detections with other methods to confirm identifications.

The *coxI* gene seems robust for species delineation in the genus *Heterodera*; however, we did observe two instances in which this marker showed potential shortcomings. The first issue was detected in our *coxI* phylogenetic analyses of the Avenae group, in which sequences of *H. arenaria* formed a clade within the larger *H. avenae* species cluster. The sequences of *H. arenaria* differ by just two bp from those of *H. avenae*, well below the intraspecific variation observed for the latter species. However, these two bp appear to be unique changes, suggesting that *H. arenaria* can at least be distinguished from *H. avenae* from a barcoding, if not a phylogenetic, perspective. This is consistent with the findings of Subbotin *et al*. (2018) where *H. arenaria* and *H. avenae* were shown to be distinct in a haplotype network but formed a polyphyly in a tree derived from Bayesian analysis. Subbotin *et al*. (2018) speculated that *H. arenaria* represents a species recently diverged from a European population of *H. avenae* and remarked that, from a phytosanitary perspective, it is best to retain the species status of *H. arenaria* because it parasitizes coastal grasses of no economic importance, rather than cereals as in *H. avenae*.

The second instance in which *coxI* showed a possible shortcoming relates to the case of distinguishing *H. carotae* from *H. cruciferae*. These two species have previously been reported as indistinguishable using PCR-ITS-RFLP profiles and ITS gene sequences (Subbotin *et al*., 2000, 2001). In a study of *H. carotae* in Mexico, Escobar-Avila *et al*. (2018) produced novel *coxI* sequences of that species but also several *coxI* sequences for *H. cruciferae*, which were the only *coxI* sequences available for the latter species at the time of writing. Escobar-Avila *et al*. (2018) found that *H. carotae* and *H. cruciferae* could not be distinguished using *coxI* sequences and concluded that to differentiate these species an integrated approach including morphology and a test of host range was necessary. Although it is entirely possible that *H. carotae* and *H. cruciferae* are indistinguishable using *coxI*, considering the former species appears restricted to carrots as hosts (Jones, 1950; Winslow, 1954; Mugniery and Bossis, 1988) and the latter to brassicas and a few species of the Lamiaceae (Winslow, 1954; Baldwin and Mundo-Ocampo, 1991), it is somewhat surprising that these species would exhibit so little divergence in such a rapidly evolving gene. Escobar-Avila *et al*. (2018) provided three sequences of *H. cruciferae*, two from specimens from the USA and one from Russia, but did not provide a morphological account of these specimens, presumably because they were all consumed in molecular analyses. The Russian sequence of *H. cruciferae* differs from those from the USA by 15 bp, whereas the USA sequences of *H. cruciferae* differ from sequences of *H. carotae* from multiple countries by as little as 1 bp. Thus, the current intraspecific variation for *H. cruciferae* is greater than the difference between some sequences of *H. carotae* and *H. cruciferae*. Because these two species are fairly similar morphologically and could easily co-occur in mixed or rotated vegetable fields, it is possible that some of the putative specimens of *H. cruciferae* used by Escobar-Avila *et al*. (2018) were misidentified. At present, there are simply too few *coxI* sequences of *H. cruciferae* to be certain of the utility of this marker, or lack thereof, for the *H. carotae*–*H. cruciferae* species pair. *Heterodera carotae* is an important pest of carrots throughout its range (Greco *et al*., 1994; Esquibet *et al*., 2020), and although *H. cruciferae* is largely considered a minor pest, there have been a few reports of significant damage to crops infested by this species (Lear *et al*., 1965; Sykes and Winfield, 1966). It is critical then that a suitable molecular marker be identified which can reliably distinguish between these two species. Using a collection of microsatellite markers, Esquibet *et al*. (2020) demonstrated a high level of genetic divergence between *H. carotae* and *H. cruciferae* and remarked that microsatellites could be used to develop a diagnostic test for these species. If further study confirms that the *coxI* gene cannot reliably distinguish between *H. carotae* and *H. cruciferae*, other molecular methods such as microsatellites (e.g., Gautier *et al*., 2019; Esquibet *et al*., 2020) can fill this diagnostic gap.

Although the number of obviously erroneous sequences detected was low, for several species of *Heterodera*, we observed high levels of intraspecific variation within one or more of the molecular markers evaluated, primarily ITS. Unsurprisingly, this was most apparent in those species/marker combinations with a large pool of sequences, such as *H. avenae*, *H. glycines*, and *H. latipons* Franklin, 1969, each of which had over 100 ITS sequences available. In these species, we observed intraspecific maxima of 80–122 bp (12%–13%) in the ITS gene. Additionally, some species had large intraspecific variation despite lower sample size, such as *H. goettingiana* with 15 ITS sequences and an intraspecific maximum of 81 bp (11%) and *H. hordecalis* with 37 ITS sequences and an intraspecific maximum of 68 bp (9%). None of these intraspecific maxima are unbelievable when considering that these datasets include sequences of individuals sourced from many geographically disparate populations (e.g., Subbotin *et al*., 2003, 2018). However, these large levels of intraspecific variation do clash with the patterns observed for most other members of the genus and notably in several other species with medium to large sequence pools, such as *H. schachtii* and *H. filipjevi*, both of which exhibit intraspecific maxima of less than 5% in ITS. We think it is likely that for several species the overall level of intraspecific variation observed for some molecular markers is inflated. In our assessment of sequence reliability, in many instances, we could not be certain if the intraspecific variation observed was due to real population-level genetic variability or artifacts of poor-quality sequencing results and/or sequence editing errors. We flagged sequences that diverged greatly from their congeners as problematic; however, in larger sequence pools, relatively small levels of variation between individual sequences become amplified, resulting in the very large levels of overall intraspecific variation detected in pairwise comparisons. This is an important limitation of our analyses and of the overall pool of publicly available sequences.

A related issue is species for which only a few sequences are available, but one or more of those sequences diverge significantly from the others. Again, it can be difficult to determine if such divergence represents natural variation or an artifact such as misidentification or sequence editing errors. Furthermore, the large number of sequences submitted to GenBank that are not associated with a published manuscript leaves many issues related to sequence identity ambiguous as there is no account of the morphology of specimens utilized. A good example of both of the above relates to the 28S sequences available for *H. cruciferae*. Sasanelli *et al*. (2013) performed a thorough study of *H. cruciferae* in Italian cabbage, identified specimens using morphology, and characterized them with ITS and 28S rDNA gene sequences. However, BLAST results of the 28S sequence of *H. cruciferae* from that study suggests that it is probably representative of *H. schachtii*, another cyst nematode widespread on brassicas (Subbotin *et al*., 2010b). There is one other 28S sequence of *H. cruciferae* available, but it is not associated with a published manuscript (KP114546; Jabbari *et al*., unpublished); this sequence is very close to *H. carotae* but not identical. This suggests that this “unpublished” sequence is accurate, but without a morphological account or other sequences with which to compare, it is still uncertain. This results in a situation where, although two 28S sequences are available for *H. cruciferae*, we still cannot be confident that either are truly representative of that species. Thirty species of *Heterodera* lack molecular data and thus cannot be diagnosed using barcoding methodologies. Of the 57 species of *Heterodera* for which molecular data are available, eight are represented by just a single sequence and over half of the remainder have just one sequence for one or more of the markers evaluated here. Where only a single sequence of a particular marker is available, it is best treated with caution when used for diagnostic purposes as additional sequences, ideally from independent studies, are needed for confidence that such sequences are truly representative of the species they are purported to be of.

There is little doubt that erroneous sequences we observed on GenBank were uploaded in good faith. However, to avoid errors, it is important that authors carefully compare their novel sequences with those available in public databases via BLAST or other phylogenetic methods before upload. Original sequencing results should be scrutinized for base-call quality, and poor-quality sequences should be discarded. Where possible, multiple sequence replicates of each gene should be produced and compared prior to upload to ensure base calls are consistent. It is also advisable to sequence multiple gene regions from individual isolates to ensure that molecular based identification is consistent across markers. We also encourage authors who have uploaded sequences which later prove to be mislabeled or problematic to correct them.

High-throughput sequencing techniques utilizing new and more accurate markers are already superseding other molecular-based diagnostic methods for cyst nematodes in many laboratories (e.g., Gautier *et al*., 2019; Esquibet *et al*., 2020). Despite this, we foresee the four markers evaluated here remaining in use for identification of plant parasitic nematodes for many years to come. With that in mind, of the four markers evaluated, *coxI* shows the greatest utility for identification and delineation of species of *Heterodera*. However, the *cox1* gene might not be best for phylogenetic interpretation (Subbotin et al., 2015, 2018); so, going forward, a combination of *coxI* and ITS, plus 28S where possible, seems ideal, especially as *coxI* data are presently available for only around a third of species of *Heterodera*. There is a real possibility of hybridization between species of *Heterodera* (Potter and Fox, 1965); thus, a combination of a mitochondrial and nuclear marker is recommended for areas where the range of multiple species overlap. Molecular diagnoses should be based not only on multiple molecular markers used in concert but also on a combination of sequence matching and tree-based phylogenetic methods. Lastly, it is important to keep in mind that no one technique is likely to be a panacea for the identification of species of *Heterodera*. New species are being described at a fairly steady rate (e.g., Li *et al*., 2020; Phougeishangbam *et al*., 2020; Jiang *et al*., 2022), and sequence data continue to accumulate and provide further insight into the population genetics, host associations, and phylogeography of *Heterodera* (Subbotin *et al*., 2018; Esquibet *et al*., 2020; Oro and Tabakovic, 2020). It is telling that so few sequences have been generated from countries in South America and Sub-Saharan Africa as this seems an obvious result of lack of resources, rather than lack of *Heterodera* species richness. Thus, there is still a great need for traditionally trained taxonomists and diagnosticians that can employ a range of techniques to identify these problematic nematodes.

## Figures and Tables

**Table S1:** Total number of sequences of species of Heterodera per country available on the NCBI database GenBank, up to 10 August 2021.

	Number of sequences recorded				
	ITS	18S	28S	*coxI*	Total
Afghanistan			1		1
Algeria	62	7	8		77
Australia	11		1	6	18
Azerbaijan	36			1	37
Belgium	21	1	2	14	38
Canada	19	1	7	5	32
Chile			1		1
China	761	16	131	21	937
Costa Rica	1		1	1	3
Cyprus	21				21
Czech Republic	7	7	7		21
Egypt	7			3	10
Estonia	2				2
France	11			9	20
Georgia	1			1	2
Germany	35		1	28	64
Ghana	2				2
Greece	6	1	1	7	15
Guatemala	2				2
India	26		15	1	42
Iran	41		16	51	108
Iraq	3			8	11
Ireland	1	2			3
Israel	7				7
Italy	19	6	8	12	45
Ivory Coast	1				1
Japan	47		64	50	161
Jordan	2		3		5
Kazakhstan	53				53
Kyrgyzstan	32			1	33
Madagascar	2				2
Mexico	3			3	6
Morocco	34			5	39
Myanmar			2		2
Netherlands	16	2	3	7	28
New Zealand	2		2	7	11
Norway				4	4
Pakistan	2		1		3
Poland	1			2	3
Portugal	1		1		2
Qatar	1				1
Russia	12			23	35
Saudi Arabia	23		1	1	25
Serbia	7			3	10
Slovak Republic	3			4	7
South Africa	3		1	1	5
South Korea	30		38	36	104
Spain	10	3	3	13	29
Sweden	4	2		2	8
Switzerland				3	3
Syria	15		1	21	37
Tajikistan	1			3	4
Tunisia	4	1	2	6	13
Turkey	219			53	272
Ukraine	3		1	7	11
United Kingdom	11		1	9	21
USA	71	7	18	158	254
Vietnam			2	1	3
Unknown	2	21	1	1	25
Total	1724	77	346	592	2739

**Table S2:** Species of Heterodera, and associated number of sequences, recorded from 59 countries based on data from the NCBI database GenBank, up to 10 August 2021.

		Total number of sequences recorded			
Species	Country	18S	ITS	28S	coxI
*Heterodera arenaria*	Italy		1		3
	Netherlands				2
	United Kingdom		3		1
*Heterodera aucklandica*	Belgium		1		
	New Zealand		1	1	3
	United Kingdom		1		2
*Heterodera australis*	Australia		7		5
	China		11		
*Heterodera avenae*	Algeria	4		6	
	Australia			1	
	Azerbaijan		9		
	China	4	359	57	4
	Czech Republic	7	7	7	
	Egypt		5		3
	France		9		7
	Germany		3		4
	India		4		1
	Iran		2	1	4
	Iraq		3		
	Israel		3		4
	Morocco		27		5
	Norway				4
	Pakistan		1		
	Saudi Arabia		23	1	1
	Serbia		1		1
	South Korea		1	2	1
	Spain		3		3
	Switzerland				1
	Syria		3		6
	Tunisia		1		6
	Turkey		39		26
	United Kingdom		1		
	USA	1	10	3	14
	Unknown	4			
*Heterodera betae*	Belgium		1		
	Germany		3		
	Netherlands		3	2	2
	Unknown	2			
*Heterodera bifenestra*	Belgium		2		
	Sweden	1	1		
*Heterodera cajani*	India		12	13	
*Heterodera cardiolata*	Pakistan		1	1	
	United Kingdom				1
*Heterodera carotae*	Belgium				1
	Algeria		2		
	Canada		5	6	5
	France		1		2
	Italy		3	1	5
	Mexico		2		1
	South Africa		1	1	1
	Switzerland				1
*Heterodera ciceri*	Syria		3		1
*Heterodera circeae*	Germany		1		
*Heterodera cruciferae*	Algeria		5		
	Iran		1	1	
	Italy		1	1	
	Netherlands		1		
	Russia		1		1
	Serbia		1		
	USA	1			2
*Heterodera cyperi*	Spain		1		
	USA		1		1
*Heterodera daverti*	Germany		3		2
	Italy		1	1	1
	Netherlands		1		1
*Heterodera dunensis*	Spain	3	3	3	8
*Heterodera elachista*	China	1	27	10	
	Iran		2	1	1
	Italy	2	4	4	2
	Japan		11	3	
*Heterodera fici*	Canada		2		
	Georgia		1		1
	Greece				4
	Iran		1		3
	Italy		2	1	
	Qatar		1		
	USA	2	1		1
*Heterodera filipjevi*	Algeria		2		
	Azerbaijan		20		
	China		58	7	2
	Germany		2		1
	Greece				1
	India		1		
	Iran		5		19
	Italy		1		
	Kazakhstan		25		
	Kyrgyzstan		31		
	Russia		3		7
	Serbia		1		2
	Slovakia		1		
	Spain		1		2
	Sweden	1			1
	Syria				6
	Tajikistan		1		3
	Turkey		80		20
	Ukraine				3
	United Kingdom		1		3
	USA		3	1	5
	Unknown	2			
*Heterodera glycines*	Canada	1	12	1	
	Chile			1	
	China	5	275	29	
	Iran		2	1	
	Japan		6	10	
	South Korea USA	1	4 21	7 7	81
	Unknown		1	1	
*Heterodera goettingiana*	China	1	10	1	3
	Germany		2	1	
	Iran		1		
	Ireland		1		
	Italy		2		
	Netherlands	4			
	USA		1		
*Heterodera goldeni*	Egypt	2			
	Iran	2			
	Israel	1			
*Heterodera graminis*	India		1		
*Heterodera graminophila*	USA	1			
*Heterodera guangdongensis*	China		3	3	
	Myanmar			2	
	Vietnam			2	1
*Heterodera hainanensis*	China		1	1	
*Heterodera hordecalis*	Algeria	2	21	2	
	Estonia		1		
	Germany		1		2
	Iran		4		3
	Israel		3		2
	Italy		2		
	Netherlands	1	2		
	Slovakia		1		2
	Sweden		1		1
	Tunisia		1		
	United Kingdom		1		
*Heterodera humuli*	Belgium				1
	Germany		1		2
	Iran		3	1	8
	Kyrgyzstan		1		1
	Portugal		1		
	Russia		1		2
	USA		6		5
*Heterodera koreana*	China	1	2	2	
	Iran		1	1	
	Japan		20	41	41
	South Korea		3	3	3
	USA	2	3	1	
*Heterodera latipons*	Cyprus		21		
	Iran		3		3
	Israel				2
	Jordan		2	3	
	Kazakhstan		28		
	Morocco		3		
	Russia		1		1
	Syria		9	1	7
	Turkey		87		3
*Heterodera litoralis*	New Zealand		1	1	4
*Heterodera mani*	Germany		4		1
	United Kingdom		1		
	USA		1		4
	Unknown	1			
*Heterodera medicaginis*	Russia		1		
	USA		13	1	14
*Heterodera mediterranea*	Italy	2	2		
	Spain		2		
	Tunisia	1	2	2	
*Heterodera microulae*	China		1	1	1
*Heterodera mothi*	Azerbaijan		1		1
	Iran		1		1
*Heterodera orientalis*	Guatemala		2		
	Russia		1		
	USA		2	1	
*Heterodera oryzae*	South Korea		1	1	1
*Heterodera oryzicola*	India		1	1	
*Heterodera persica*	Iran		1		
*Heterodera pratensis*	Belgium				1
	Germany		8		8
	Iran				5
	Netherlands		1		1
	Russia		1		2
	South Korea		2	1	2
	USA		1		1
*Heterodera ripae*	Belgium Germany		1		5 4
	Greece		1		2
	Russia Serbia		1 1		4
	Slovakia				2
	Sweden		1		
	United Kingdom				2
*Heterodera sacchari*	Ghana		2		
	Ivory Coast		1		
*Heterodera salixophila*	Belgium		1	1	3
	Germany				4
	Iran		1	1	
	Russia				5
	Ukraine		3		4
*Heterodera schachtii*	Algeria		1		
	Australia		4		
	Belgium	1	15		
	China	1	1	8	3
	France		1	3	
	Germany		4		
	Iran		2		1
	Ireland	2			
	Japan		1	1	1
	Mexico		1		2
	Morocco		3		
	Netherlands		8	1	1
	Poland		1		1
	Serbia		3		
	South Africa		1		
	South Korea		3	4	7
	Turkey		12		
	USA		2	3	22
	Unknown	6	1		
*Heterodera scutellariae*	Germany		1		
*Heterodera sinensis*	China		1		
*Heterodera skohensis*	India		1		
*Heterodera sojae*	China		1	1	
	Japan		1	1	
	South Korea		3	8	12
*Heterodera sorghi*	India		1		
*Heterodera sturhani*	China				7
*Heterodera trifolii*	Costa Rica		1	1	1
	Estonia		1		
	Iran		1	1	2
	Italy				1
	Japan		8	8	8
	South Korea		8	8	9
	United Kingdom	1	2	1	
	USA		2		5
	Unknown	4			
*Heterodera turcomanica*	Iran		1		1
*Heterodera urticae*	Belgium		1	1	3
*Heterodera ustinovi*	Belgium		1		
	Germany		1		
	Slovakia		1		
	United Kingdom		1		
	USA		1		2
*Heterodera vallicola*	Russia		1		1
*Heterodera zeae*	Afghanistan			1	
	China		5		
	Greece	1	5	1	
	India		4		
	Portugal			1	
	USA		2	1	

## References

[j_jofnem-2022-0024_ref_001] Abad P., Gouzy J., Aury J.-M., Castagnone-Sereno P., Danchin E. G., Deleury E., Perfus-Barbeoch L., Anthouard V., Artiguenave F., Blok V. C. (2008). Genome sequence of the metazoan plant-parasitic nematode Meloidogyne incognita. Nature Biotechnology.

[j_jofnem-2022-0024_ref_002] Abdelfattah A., Malacrino A., Wisniewski M., Cacciola S. O., Schena L. (2018). Metabarcoding: A powerful tool to investigate microbial communities and shape future plant protection strategies. Biological Control.

[j_jofnem-2022-0024_ref_003] Altschul S. F., Gish W., Miller W., Myers E. W., Lipman D. J. (1990). Basic local alignment search tool. Journal of Molecular Biology.

[j_jofnem-2022-0024_ref_004] Baldwin J. G. (1992). Evolution of cyst and non-cyst-forming Heteroderinae. Annual Review of Phytopathology.

[j_jofnem-2022-0024_ref_005] Baldwin J. G., Mundo-Ocampo M., Nickle W. R. (1991). Manual of agricultural nematology.

[j_jofnem-2022-0024_ref_006] Bert W., Leliaert F., Vierstraete A. R., Vanfleteren J. R., Borgonie G. (2008). Molecular phylogeny of the Tylenchina and evolution of the female gonoduct (Nematoda: Rhabditida). Molecular Phylogenetics and Evolution.

[j_jofnem-2022-0024_ref_007] Boag B., Yeates G. W. (1998). Soil nematode biodiversity in terrestrial ecosystems. Biodiversity & Conservation.

[j_jofnem-2022-0024_ref_008] Coomans A. (2002). Present status and future of nematode systematics. Nematology.

[j_jofnem-2022-0024_ref_009] Edgar R. C. (2004). MUSCLE: Multiple sequence alignment with high accuracy and high throughput. Nucleic Acids Research.

[j_jofnem-2022-0024_ref_010] Escobar-Avila I. M., López-Villegas E. Ó., Subbotin S. A., Tovar-Soto A. (2018). First report of carrot cyst nematode Heterodera carotae in Mexico: Morphological, molecular characterization, and host range study. Journal of Nematology.

[j_jofnem-2022-0024_ref_011] Esquibet M., Gautier C., Piriou C., Grenier E., Fournet S., Montarry J. (2020). Evidence of strong gene flow among French populations of the carrot cyst nematode Heterodera carotae. Plant Pathology.

[j_jofnem-2022-0024_ref_012] Eyualem-Abebe J. G. B., Adams B., Hope D., Gardner S., Huettel R., Mullin P., Powers T., Sharma J., Ye W., Thomas W. K. (2006). A position paper on the electronic publication of nematode taxonomic manuscripts. Journal of Nematology.

[j_jofnem-2022-0024_ref_013] Ferris V. R., Ferris J. M., Faghihi J. (1993). Variation in spacer ribosomal DNA in some cyst-forming species of plant parasitic nematodes. Fundamental and Applied Nematology.

[j_jofnem-2022-0024_ref_014] Ferris V. R., Sabo A., Baldwin J. G., Mundo-Ocampo M., Inserra R. N., Sharma S. (2004). Phylogenetic relationships among selected Heteroderoidea based on 18S and ITS ribosomal DNA. Journal of Nematology.

[j_jofnem-2022-0024_ref_015] Gautier C., Esquibet M., Fournet S., Piriou C., Yvin J.-C., Nguema-Ona E., Grenier E., Montarry J. (2019). Microsatellite markers reveal two genetic groups in European populations of the carrot cyst nematode Heterodera carotae. Infection, Genetics and Evolution.

[j_jofnem-2022-0024_ref_016] Giebner H., Langen K., Bourlat S. J., Kukowka S., Mayer C., Astrin J. J., Misof B., Fonseca V. G. (2020). Comparing diversity levels in environmental samples: DNA sequence capture and metabarcoding approaches using 18S and COI genes. Molecular Ecology Resources.

[j_jofnem-2022-0024_ref_017] Greco N., D’addabbo T., Brandonisio A., Elia F. (1994). Damage to Italian crops caused by cyst-forming nematodes. Journal of Nematology.

[j_jofnem-2022-0024_ref_018] Hardulak L. A., Morinière J., Hausmann A., Hendrich L., Schmidt S., Doczkal D., Müller J., Hebert P. D., Haszprunar G. (2020). DNA metabarcoding for biodiversity monitoring in a national park: Screening for invasive and pest species. Molecular Ecology Resources.

[j_jofnem-2022-0024_ref_019] Hodda M. (2022). Phylum Nematoda: A classification, catalogue and index of valid genera, with a census of valid species. Zootaxa.

[j_jofnem-2022-0024_ref_020] Jiang R., Hu X., Li Y., Bian Y., Huang L., Gu J., Liu P., Huang W., Kong L., Liu S., Peng H., Peng D. (2022). Heterodera amaranthusiae n. sp. Nematoda: Heteroderidae), a new cyst nematode parasitising Amaranthus retroflexus L. in China. Nematology.

[j_jofnem-2022-0024_ref_021] Jones F. G. W. (1950). Observations on the beet eelworm and other cyst‐forming species of Heterodera. Annals of Applied Biology.

[j_jofnem-2022-0024_ref_022] Katoh K., Rozewicki J., Yamada K. D. (2019). MAFFT online service: Multiple sequence alignment, interactive sequence choice and visualization. Briefings in Bioinformatics.

[j_jofnem-2022-0024_ref_023] Koenning S. R., Overstreet C., Noling J. W., Donald P. A., Becker J. O., Fortnum B. A. (1999). Survey of crop losses in response to phytoparasitic nematodes in the United States for 1994. Journal of Nematology.

[j_jofnem-2022-0024_ref_024] Kumar S., Stecher G., Li M., Knyaz C., Tamura K. (2018). MEGA X: Molecular evolutionary genetics analysis across computing platforms. Molecular Biology and Evolution.

[j_jofnem-2022-0024_ref_025] Lear B., Johnson D. E., Miyogawa S. T., Sciaroni R. H. (1965). Response of Brussels sprouts to soil fumigation for control of cabbage root nematode, Heterodera cruciferae. Plant Disease Reporter.

[j_jofnem-2022-0024_ref_026] Li W., Li H., Ni C., Peng D., Liu Y., Luo N., Xu X. (2020). Description of Heterodera microulae sp. n. (Nematoda: Heteroderinae) from China – A new cyst nematode in the Goettingiana group. Journal of Nematology.

[j_jofnem-2022-0024_ref_027] Luc M., Weischer R., Stone A., Baldwin J. G. (1986). On the definition of heteroderid cysts. Revue de Nématologie.

[j_jofnem-2022-0024_ref_028] Macheriotou L., Guilini K., Bezerra T. N., Tytgat B., Nguyen D. T., Phuong Nguyen T. X., Noppe F., Armenteros M., Boufahja F., Rigaux A. (2019). Metabarcoding free‐living marine nematodes using curated 18S and CO1 reference sequence databases for species‐level taxonomic assignments. Ecology and Evolution.

[j_jofnem-2022-0024_ref_029] Meagher J. W. (1982). Yield loss caused by Heterodera avenae in cereal crops grown in a Mediterranean climate. Eppo Bulletin.

[j_jofnem-2022-0024_ref_030] Mugniery D., Bossis M. (1988). Heterodera carotae Jones, 1950. 1. Gamme d’hôtes, vitesse de développement, cycle. Revue de Nématologie.

[j_jofnem-2022-0024_ref_031] Mundo-Ocampo M., Troccoli A., Subbotin S., Del Cid J., Baldwin J., Inserra R. (2008). Synonymy of Afenestrata with Heterodera supported by phylogenetics with molecular and morphological characterisation of H. koreana comb. n. and H. orientalis comb. n. (Tylenchida: Heteroderidae). Nematology.

[j_jofnem-2022-0024_ref_032] Nicol J. M., Elekçioğlu I. H., Bolat N., Rivoal R. (2007). The global importance of the cereal cyst nematode (Heterodera spp.) on wheat and international approaches to its control. Communications in Agricultural and Applied Biological Sciences.

[j_jofnem-2022-0024_ref_033] Nicol J. M., Rivoal R., Ciancio A., Mukerji K. G. (2008). Integrated management and biocontrol of vegetable and grain crops nematodes.

[j_jofnem-2022-0024_ref_034] Nicol J. M., Turner S. J., Coyne D. L., den Nijs L. J. M. F., Hockland S., Maafi Z. T., Jones J., Gheysen G., Fenoll C. (2011). Genomics and molecular genetics of plant-nematode interactions.

[j_jofnem-2022-0024_ref_035] Oliveira C. M. G. d., Monteiro A. R., Blok V. C. (2011). Morphological and molecular diagnostics for plant-parasitic nematodes: Working together to get the identification done. Tropical Plant Pathology.

[j_jofnem-2022-0024_ref_036] Oro V., Tabakovic M. (2020). Phylogeography of some European populations of the sugar beet cyst nematode. Russian Journal of Nematology.

[j_jofnem-2022-0024_ref_037] Palomares-Rius J. E., Cantalapiedra-Navarrete C., Castillo P. (2014). Cryptic species in plant-parasitic nematodes. Nematology.

[j_jofnem-2022-0024_ref_038] Phani V., Khan M. R., Dutta T. K. (2021). Plant-parasitic nematodes as a potential threat to protected agriculture: Current status and management options. Crop Protection.

[j_jofnem-2022-0024_ref_039] Phougeishangbam R. S., Karssen G., Couvreur M., Bert W. (2020). Morphological and molecular characterization of Heterodera dunensis n. sp. (Nematoda: Heteroderidae) from Gran Canaria, Canary Islands. Journal of Nematology.

[j_jofnem-2022-0024_ref_040] Potter J. W., Fox J. A. (1965). Hybridization of Heterodera schachtii and H. glycines. Phytopathology,.

[j_jofnem-2022-0024_ref_041] Powers T., Skantar A., Harris T., Higgins R., Mullin P., Hafez S., Handoo Z., Todd T., Powers K. (2019). DNA barcoding evidence for the North American presence of alfalfa cyst nematode, Heterodera medicaginis. Journal of Nematology.

[j_jofnem-2022-0024_ref_042] Procter D. L. C. (1984). Towards a biogeography of free-living soil nematodes. I. Changing species richness, diversity and densities with changing latitude. Journal of Biogeography.

[j_jofnem-2022-0024_ref_043] Ruppert K. M., Kline R. J., Rahman M. S. (2019). Past, present, and future perspectives of environmental DNA (eDNA) metabarcoding: A systematic review in methods, monitoring, and applications of global eDNA. Global Ecology and Conservation.

[j_jofnem-2022-0024_ref_044] Salas A., Barrera M. D., Achinelly M. F. (2022). Abundance, diversity, and distribution of plant-parasitic nematodes in horticultural soils under different management systems in a tomato-producing area in Argentina. Nematology.

[j_jofnem-2022-0024_ref_045] Sasanelli N., Vovlas N., Trisciuzzi N., Cantalapiedra-Navarrete C., Palomares-Rius J. E., Castillo P. (2013). Pathogenicity and host–parasite relationships of Heterodera cruciferae in cabbage. Plant Disease.

[j_jofnem-2022-0024_ref_046] Sekimoto S., Uehara T., Mizukubo T. (2017). Characterisation of populations of Heterodera trifolii Goffart, 1932 (Nematoda: Heteroderidae) in Japan and their phylogenetic relationships with closely related species. Nematology.

[j_jofnem-2022-0024_ref_047] Siddiqi M. R. (2000). Tylenchida: Parasites of plants and insects.

[j_jofnem-2022-0024_ref_048] Singh S. K., Hodda M., Ash G. J. (2013). Plant‐parasitic nematodes of potential phytosanitary importance, their main hosts and reported yield losses. Eppo Bulletin.

[j_jofnem-2022-0024_ref_049] Singh S., Singh B., Singh A. P. (2015). Nematodes: A threat to sustainability of agriculture. Procedia Environmental Sciences.

[j_jofnem-2022-0024_ref_050] Smiley R. W., Dababat A. A., Iqbal S., Jones M. G. K., Maafi Z. T., Peng D., Subbotin S. A., Waeyenberge L. (2017). Cereal cyst nematodes: A complex and destructive group of Heterodera species. Plant Disease.

[j_jofnem-2022-0024_ref_051] Smiley R. W., Whittaker R. G., Gourlie J. A., Easley S. A., Ingham R. E. (2005). Plant-parasitic nematodes associated with reduced wheat yield in Oregon: Heterodera avenae. Journal of Nematology.

[j_jofnem-2022-0024_ref_052] Subbotin S., Sturhan D., Moens M. (2003). Molecular and morphological characterisation of the Heterodera avenae species complex (Tylenchida: Heteroderidae). Nematology.

[j_jofnem-2022-0024_ref_053] Subbotin S., Waeyenberge L., Moens M. (2000). Identification of cyst forming nematodes of the genus Heterodera (Nematoda: Heteroderidae) based on the ribosomal DNA-RFLP. Nematology.

[j_jofnem-2022-0024_ref_054] Subbotin S. A., Akanwari J., Nguyen C. N., del Prado Vera I. C., Chitambar J. J., Inserra R. N., Chizhov V. N. (2017). Molecular characterisation and phylogenetic relationships of cystoid nematodes of the family Heteroderidae (Nematoda: Tylenchida). Nematology.

[j_jofnem-2022-0024_ref_055] Subbotin S. A., Mundo-Ocampo M., Baldwin J. G. (2010a). Systematics of cyst nematodes (Nematoda: Heteroderinae).

[j_jofnem-2022-0024_ref_056] Subbotin S. A., Mundo-Ocampo M., Baldwin J. G. (2010b). Systematics of cyst nematodes (Nematoda: Heteroderinae), Part B. Leiden.

[j_jofnem-2022-0024_ref_057] Subbotin S. A., Stanley J. D., Ploeg A. T., Maafi Z. T., Tzortzakakis E. A., Chitambar J. J., Palomares-Rius J. E., Castillo P., Inserra R. N. (2015). Characterisation of populations of Longidorus orientalis Loof, 1982 (Nematoda: Dorylaimida) from date palm (Phoenix dactylifera L.) in the USA and other countries and incongruence of phylogenies inferred from ITS1 rRNA and coxI genes. Nematology.

[j_jofnem-2022-0024_ref_058] Subbotin S. A., Sturhan D., Rumpenhorst H. J., Moens M. (2002). Description of the Australian cereal cyst nematode Heterodera australis sp. n. (Tylenchida: Heteroderidae). Russian Journal of Nematology.

[j_jofnem-2022-0024_ref_059] Subbotin S. A., Toumi F., Elekçioğlu I. H., Waeyenberge L., Maafi Z. T. (2018). DNA barcoding, phylogeny and phylogeography of the cyst nematode species of the Avenae group from the genus Heterodera (Tylenchida: Heteroderidae). Nematology.

[j_jofnem-2022-0024_ref_060] Subbotin S. A., Vierstraete A., De Ley P., Rowe J., Waeyenberge L., Moens M., Vanfleteren J. R. (2001). Phylogenetic relationships within the cyst-forming nematodes (Nematoda, Heteroderidae) based on analysis of sequences from the ITS regions of ribosomal DNA. Molecular Phylogenetics and Evolution.

[j_jofnem-2022-0024_ref_061] Sykes G. B., Winfield A. L. (1966). Studies on brassica cyst nematode Heterodera cruciferae. Nematologica.

[j_jofnem-2022-0024_ref_062] Szalanski A. L., Sui D. D., Harris T. S., Powers T. O. (1997). Identification of cyst nematodes of agronomic and regulatory concern with PCR-RFLP of ITS1. Journal of Nematology.

[j_jofnem-2022-0024_ref_063] Tanha Maafi Z., Subbotin S., Moens M. (2003). Molecular identification of cyst-forming nematodes (Heteroderidae) from Iran and a phylogeny based on ITS-rDNA sequences. Nematology.

[j_jofnem-2022-0024_ref_064] Toumi F., Waeyenberge L., Viaene N., Dababat A., Nicol J. M., Ogbonnaya F., Moens M. (2013). Development of two species-specific primer sets to detect the cereal cyst nematodes Heterodera avenae and Heterodera filipjevi. European Journal of Plant Pathology.

[j_jofnem-2022-0024_ref_065] Valentin R. E., Fonseca D. M., Nielsen A. L., Leskey T. C., Lockwood J. L. (2018). Early detection of invasive exotic insect infestations using eDNA from crop surfaces. Frontiers in Ecology and the Environment.

[j_jofnem-2022-0024_ref_066] Vovlas N., Vovlas A., Leonetti P., Liébanas G., Castillo P., Subbotin S. A., Rius J. E. P. (2015). Parasitism effects on white clover by root-knot and cyst nematodes and molecular separation of Heterodera daverti from H. trifolii. European Journal of Plant Pathology.

[j_jofnem-2022-0024_ref_067] Waeyenberge L., Viaene N., Subbotin S. A., Moens M., Riley I. T., Nicol J. M., Dababat A. A. (2009). Cereal cyst nematodes: Status, research and outlook.

[j_jofnem-2022-0024_ref_068] Wickham H. (2009). ggplot2: Elegant graphics for data analysis.

[j_jofnem-2022-0024_ref_069] Winslow R. D. (1954). Provisional lists of host plants of some root eelworms (Heterodera spp.). Annals of Applied Biology.

[j_jofnem-2022-0024_ref_070] Young R. G., Milián‐García Y., Yu J., Bullas‐ Appleton E., Hanner R. H. (2021). Biosurveillance for invasive insect pest species using an environmental DNA metabarcoding approach and a high salt trap collection fluid. Ecology and Evolution.

